# Perioperative risk factors for serious gastrointestinal complications treated by laparotomy after cardiac surgery using cardiopulmonary bypass

**DOI:** 10.1186/cc9426

**Published:** 2011-03-11

**Authors:** P Soos, B Schmack, A Weymann, G Veres, B Merkely, M Karck, G Szabó

**Affiliations:** 1 Semmelweis University, Budapest, Hungary; 2 University of Heidelberg, Germany

## Introduction

Gastrointestinal (GI) complications are rare but often fatal consequences of cardiac surgery, especially after cardiopulmonary bypass (CPB) operations. The therapy can be conservative or - in critical cases - surgical; however, an early and safe diagnosis may prevent the development of life-threatening GI complications. The aim of our study was to characterize the risk factors and perioperative predictors for GI complications treated by laparotomy after CPB operations.

## Methods

In a retrospective analysis of 12 years of CPB operations, 13,553 consecutive patients were involved in the study. Laparotomy was performed after CPB in 277 (2.01%) cases, the mean follow-up time was 63.9 months.

## Results

Logistic regression analysis of the preoperative data demonstrated RR = 1.585 (OR: 1.340 to 1.876, *P *< 0.001) for heart failure according to the NYHA classification. The postoperative data analysis showed an RR = 12.257 (OR: 9.604 to 15.643, *P *< 0.001) for the need of an IABP implantation and an RR = 13.455 (OR: 10.516 to 17.215, *P *< 0.001) of low output syndrome in the GI complications group. In contrast, GI disease in the patient history seemed not to be a significant risk factor. Preoperative renal failure had an RR = 2.181 (OR: 1.686 to 2.821, *P *< 0.001) until postoperative renal failure had an RR = 29.145 (OR: 21.322 to 39.839, *P *< 0.001).

## Conclusions

A failing heart may play a significant role in critical GI complications after CPB, whereas history of GI disease does not seem to determine its incidence.

